# OprD Repression upon Metal Treatment Requires the RNA Chaperone Hfq in *Pseudomonas aeruginosa*

**DOI:** 10.3390/genes7100082

**Published:** 2016-10-03

**Authors:** Verena Ducret, Manuel R. Gonzalez, Tiziana Scrignari, Karl Perron

**Affiliations:** 1 Microbiology Unit, Department of Botany and Plant Biology, Sciences III, University of Geneva, Geneva 1211, Switzerland; verena.ducret@unige.ch (V.D.); manuel.gonzalez@unige.ch (M.R.G.); 2 EPFL-SV-GHI-UPBLO, Lausanne 1015, Switzerland; tiziana.scrignari@epfl.ch

**Keywords:** Hfq, carbapenem resistance, *Pseudomonas aeruginosa*, OprD, two-component system, Zinc, Copper

## Abstract

The metal-specific CzcRS two-component system in *Pseudomonas aeruginosa* is involved in the repression of the OprD porin, causing in turn carbapenem antibiotic resistance in the presence of high zinc concentration. It has also been shown that CzcR is able to directly regulate the expression of multiple genes including virulence factors. CzcR is therefore an important regulator connecting (i) metal response, (ii) pathogenicity and (iii) antibiotic resistance in *P. aeruginosa*. Recent data have suggested that other regulators could negatively control *oprD* expression in the presence of zinc. Here we show that the RNA chaperone Hfq is a key factor acting independently of CzcR for the repression of *oprD* upon Zn treatment. Additionally, we found that an Hfq-dependent mechanism is necessary for the localization of CzcR to the *oprD* promoter, mediating *oprD* transcriptional repression. Furthermore, in the presence of Cu, CopR, the transcriptional regulator of the CopRS two-component system also requires Hfq for *oprD* repression. Altogether, these results suggest important roles for this RNA chaperone in the context of environment-sensing and antibiotic resistance in *P. aeruginosa*.

## 1. Introduction

*Pseudomonas aeruginosa* is an opportunistic pathogen that causes serious and diverse infections in host organisms by producing a broad range of virulence factors [1]. This bacterium carries intrinsic resistances to multiple classes of antimicrobial compounds, representing a major challenge for the treatment of *Pseudomonas*’ infections [2]. For instance, resistance to carbapenem, an important class of anti-*Pseudomonas* compounds, is mostly caused by the decrease in production of OprD porin. In normal conditions, OprD forms a trimeric outer-membrane channel [3] which is generally involved in the import of basic amino acids and small peptides from the outer medium [4]. However, carbepenem molecules are also imported through this porin and, consequently, a reduced production of OprD causes the insurgence of bacterial resistance [5,6,7].

We have previously found that the mechanism that triggers the negative regulation of OprD is linked to Zn and Cd metal resistance. According to [8], this mechanism is a process called co-regulation between metal and antibiotic resistance. The presence of an excess of these elements activates the metal-inducible CzcRS two-component system (TCS) that induces the expression of a metal efflux pump. Furthermore, it down-regulates the production of the OprD porin, thus rendering cells resistant to both trace metals and carbapenems. Cu has also been shown to induce expression of the *copRS* TCS which can directly repress *oprD* transcription [5]. Therefore, toxic metal concentrations of Zn, Cd, or Cu may all lead to the induction of carbapenem resistance. In addition to OprD, the CzcR regulator has been shown to modulate gene expression of multiple virulence factors in response to Zn treatment, with major clinical implications [9]. Consistently, certain physiological environments enriched in metals, such as the pulmonary sputum of cystic fibrosis (CF) patients, can increase both the virulence and carbapenem resistance of *P. aeruginosa* [10]. These situations could locally induce carbapenem resistance, rendering this antibiotic inefficient and could explain, in part, the discrepancies between antibiotic susceptibility profiles performed in vitro and effective resistance profiles in patients.

*P. aeruginosa* possesses a broad range of TCS systems, allowing it to adapt and thrive in many diverse environments by specifically modulating the transcription of response genes [11]. In addition to TCS signaling and transcriptional adaptation, bacteria take advantage of post-transcriptional regulation mechanisms to adjust their cellular functions. Several studies have shown that the expression of porins in *Escherichia coli* [12], *Vibrio cholerae* [13] and *Salmonella* [14,15] is regulated by the Hfq protein in association with specific small non-coding RNAs (sRNA) [16,17]. Hfq interacts with specific sRNAs and facilitates the binding to their target-mRNA, allowing the direct modulation of translation or of mRNA stability [17,18]. The Hfq protein is therefore a key player in the post-transcriptional regulation process involving sRNA. Hfq belongs to the Sm family of proteins, which members are found in bacteria, eukaryotes and archea [19]. Its crystal structure, characterized by a ring-like structure composed of six monomer subunits, has been solved for several bacteria such as *Staphylococcus aureus* [20], *Bacillus subtilis* [21], *E. coli* [22] and *P. aeruginosa* [23,24]. It has been shown to affect the expression of up to 5% of *P. aeruginosa* transcripts, mainly through post-transcriptional regulations [25]. Hfq deletion mutants present diminished fitness, growth defects and impaired resistance under conditions of stress. Interestingly, in pathogenic bacteria, the loss of Hfq reduces virulence in in vivo models [26] and impairs quorum sensing capacities [27].

In this study, we investigated the regulatory network controlling OprD porin production in *P. aeruginosa* in the presence of high Zn concentrations. Our results show that Hfq is required for OprD downregulation upon Zn treatment. More precisely, we found that the DNA-binding activity of the CzcR protein to the *oprD* promoter is strongly affected in an *hfq* mutant. Moreover, the CopRS TCS involved in copper response also requires Hfq to repress the porin. Altogether, these data highlight an important function for the RNA chaperone Hfq in the control of TCS signaling and might help the understanding of the induction of metal and antibiotic resistance in the environment. This might also explain the inability of *hfq* mutants to adapt to changing environments and open new opportunities for drug design in the context of anti-Pseudomonas therapy.

## 2. Materials and Methods

### 2.1. Bacterial Strains, Growth Conditions and Minimum Inhibitory Concentration (MIC) Determination

The bacterial strains used in this study are listed in Table 1. *E. coli* and *P. aeruginosa* strains were grown at 37 °C in solid or liquid Luria-Bertani (LB) medium (US biological, Salem, MA, USA). Metal-containing LB media were supplemented with CuCl_2_ or ZnCl_2_ at the final concentration indicated in the figure legends. Antibiotics were used at the following concentrations (in µg per mL): gentamicin 50, tetracycline 50, carbenicillin 200, streptomycin 200 for *P. aeruginosa* and gentamicin 15, tetracycline 15, ampicillin 100 for *E. coli*.

The minimum inhibitory concentration (MIC) for imipenem was determined according to the EUCAST (European Committee on Antimicrobial Susceptibility Testing) procedure [28] in LB medium supplemented or not with 2.5 mM CuCl_2_ or 0.5 mM ZnCl_2,_ using the Imipenem E-test (Biomerieux, Marcy-l’Etoile, France).

### 2.2. DNA Manipulations

Restriction enzymes, PCR amplifications and cloning were performed using standard methods [31] following the manufacturers instructions. Plasmids were introduced into *E. coli* by heat-shock [31] and into *P. aeruginosa* by electroporation [32]. Plasmid inserts and genomic regions flanking the deletions were verified by sequencing.

*hfq* complementation*:* The full *P. aeruginosa* *hfq* gene and its promoter region were amplified by PCR using primers 636 and 610 (Appedix Table A1). The PCR product was digested with EcoRI and BamHI enzymes and ligated into the corresponding site of the pME4510 vector. The resulting plasmid containing the 6× His tag in the C-terminal part of the *hfq* gene was transformed into the *P. aeruginosa* WT strain and the *hfq^−^* mutant by electroporation [32].

Double mutant construction*:* The *ΔczcRS;hfq^−^* double mutant was constructed by homologous recombination. Briefly, the *ΔczcRS* strain was conjugated with the p202Sp plasmid containing the inactivated *hfq* gene, according to the initial protocol [26]. Clones were selected for streptomycin resistance and tetracycline sensitivity. Deletion was confirmed by PCR on genomic DNA using primers 314 and 315 (Appedix Table A1).

### 2.3. Western Blot Analysis

Overnight cultures were diluted to an OD_600_ of 0.05 for Wild Type (WT) and *ΔczcRS* or OD_600_ of 0.1 (absence of Zn) or 0.2 (presence of Zn) for *hfq^−^* and *ΔczcRS;hfq^−^* strains and grown for 6 h. Metal concentrations, when added, are 0.5 mM ZnCl_2_ or 2.5 mM CuCl_2_. When necessary, IPTG (isopropyl-1-thio-D-galactopyranoside) was added at a final concentration of 0.1 mM. Cultures were grown for 6 h and 1 mL of culture was pelleted in a microfuge. Total proteins were solubilized to a concentration of 2 mg·mL^−1^ by sonication in the appropriate volume of 1× β-mercaptoethanol gel-loading buffer (an OD_600_ of 1 gives 0.175 mg·mL^−1^ of protein). Samples were boiled for 5 min prior to loading. 15 µL (30 µg) of total protein was separated on a 4%–15% precast gel (Mini-PROTEAN TGX Gels, Biorad, Hercules, CA, USA) and transferred to a nitrocellulose membrane. Blots were incubated with anti-OprD, anti-CzcR and anti-Hsp70 antibodies as previously described [9] and revealed by chemiluminescence.

### 2.4. RNA Extraction and Reverse Transcription

Overnight cultures were diluted as described in Section 2.3. and grown for 6 h. RNA Protect bacteria solution (1 mL, Qiagen, Hilden, Germany) was added to 0.5 mL of culture. Cells were harvested by centrifugation and total RNA was extracted using RNeasy columns (Qiagen) according to the manufacturer’s instructions. RNA (3 µg) was treated for 2 h with RQ1 DNase (Promega, Fitchburg, MA, USA), in order to remove any residual DNA, followed by phenol-chloroform extraction and ethanol precipitation. The RNA was then resuspended in 30 μL RNAse-free water. For cDNA synthesis, 500 ng of total RNA was reverse-transcribed using random hexamer primers (Promega) and Improm-II reverse transcriptase (Promega) according to the supplier’s instructions. The reverse transcriptase was then heat-inactivated and the cDNAs obtained were diluted tenfold in water.

### 2.5. Quantitative RT-PCR

qPCR procedures were performed in triplicate starting from three independent experiments, using SYBR Green mix (Power SYBR Green PCR Master Mix, Thermo Fisher Scientific, Waltham, MA, USA), according to the manufacturer’s instructions. Primers used for qRT-PCR are described in Appedix Table A1. Results were analyzed according to [33]. The well expressed porin *oprF* was used for normalization since its expression was affected neither by Zn nor by *hfq* deletion.

### 2.6. Semi-Quantitative RT-PCR

PCR amplifications were performed using standard procedures with 27 cycles, except for the RT-negative control (without reverse-transcriptase) for which the amplification was carried out with 30 cycles, using *hsp70* primer pairs. The primers used are listed in Appedix Table A1. Each analysis was performed at least three times from three independent cultures. A representative analysis is presented.

### 2.7. Chromosome Immunoprecipitation

ChIP (chromosome immunoprecipitation) experiments were performed as previously described [9]. Briefly, the WT and *hfq^−^* strains were grown for 6 h in 50 mL LB medium supplemented or not with 0.5 mM Zn. To fix the protein to DNA, the cultures were treated with 1.2% (final concentration) formaldehyde for 10 min. Glycine (330 mM) was then added to quench the reaction. Bacteria were lyzed by sonication and resuspended in 500 µL ice-cold FA-lysis buffer (50 mM HEPES-KOH pH 7.5, 140 mM NaCl, 1 mM EDTA pH 8, 1% Triton X-100, 0.1% Sodium deoxycholate) supplemented with lysozyme (5 mg·mL^−1^), AEBSF (4-(2-aminoethyl) benzenesulfonyl fluoride hydrochloride, 1 mM final) and SDS (0.5% final). The resuspended pellet was sonicated as previously described [34]. After sample centrifugation 200 µL of supernatant was used for immunoprecipitation. The immunoprecipitations were performed by addition of 800 µL FA-lysis buffer with 50 µL protein A sepharose (100 mg·mL^−1^) and 5 µL of CzcR anti-serum [10]. Immunoprecipitated DNA was quantified by real-time PCR using a SYBR Green mix (Power SYBR Green PCR Master Mix, Thermo Fisher Scientific, Waltham, MA, USA) according to the supplier's specifications. Results represent the amount of *oprD* promoter immunoprecipitated with CzcR in comparison to the input DNA. Each value represents the average of three independent experiments (standard deviations are indicated). The primers used are listed in Appedix Table A1.

## 3. Results

### 3.1. A CzcR-Independent Mechanism Represses the Production of the OprD Porin in Presence of Zn

**Figure 1:**
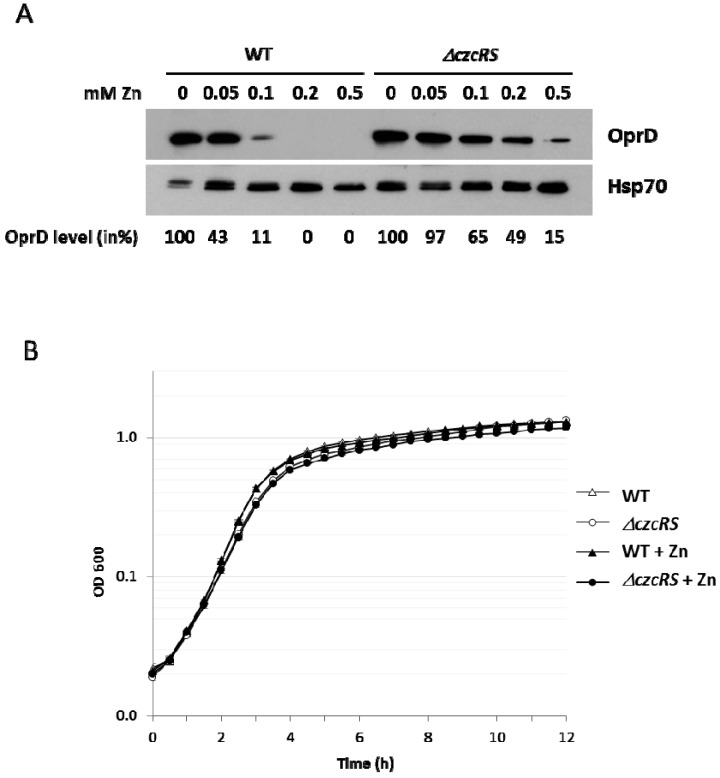
OprD is downregulated in the presence of Zn independently of CzcRS. (**A**) Immunoblot analysis of OprD porin on total protein extract of the *Pseudomonas aeruginosa* WT strain and the *ΔczcRS* mutant cultivated for 6 h in Luria-Bertani (LB) medium with increasing concentrations of ZnCl_2_ as indicated. Blots were exposed to anti-OprD and anti-Hsp70 (loading control) antibodies. OprD quantification was performed using ImageJ software (NIH, Bethesda, MD, USA). OprD band intensity was normalized to Hsp70 intensity and expressed as a percentage of the condition of the absence of Zn; (**B**) Growth curves of *P. aeruginosa* WT strain and the *ΔczcRS* mutant cultivated in LB medium in the absence or presence of 0.5 mM ZnCl_2_ (+Zn).

CzcR, the transcriptional regulator of the Zn and Cd activated two-component system CzcRS, is able to directly repress the transcription of the *oprD* gene in the presence of Zn [7,9]. In a previous study we have shown that another mechanism, independently of CzcRS, could also be involved in *oprD* repression upon Zn treatment [10]. We decided to further investigate this alternative mechanism and used the *ΔczcRS* mutant, deleted for the metal-specific TCS, to analyze the abundance of OprD porin at different Zn concentrations in LB medium when cells are in early stationary phase, i.e., after 6 h of growth (Figure 1A). In the wild type *P. aeruginosa* PAO1 strain, at 0.05 mM Zn, we observed at 6 h a drop of OprD of approximately 60%, whereas no effect (only 3% reduction) was observed in the corresponding *ΔczcRS* mutant (Figure 1A). At 0.2 mM Zn, OprD could no longer be detected by western blot in the WT. Surprisingly, at this concentration, we observed a 50% decrease in OprD protein in the *ΔczcRS* strain. By increasing Zn to 0.5 mM, a concentration that does not affect the growth of the mutant (Figure 1B), we found that OprD is also strongly repressed (85%) in the *ΔczcRS* mutant (Figure 1A)*.* Although this effect is not as strong as the one observed in the WT, it does however suggest that another mechanism, independent of CzcRS, might account for OprD down-regulation in the presence high Zn concentrations.

### 3.2. Hfq is Involved in the Repression of OprD in the Presence of Zn

**Figure 2:**
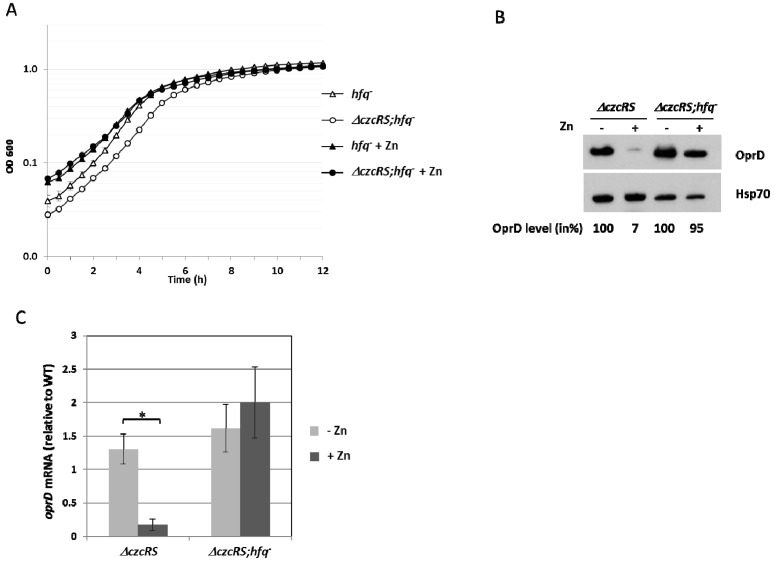
Hfq is involved in *oprD* repression in the presence of Zn. (**A**) Growth curves of *P. aeruginosa* *hfq^−^* single mutant and the *ΔczcRS;hfq^−^* double mutant cultivated in LB medium in the absence or presence of 0.5 mM ZnCl_2_ (+Zn); (**B**) Immunoblot analysis; total protein extract of the *ΔczcRS* and the *ΔczcRS;hfq^−^* mutants cultivated in the absence or presence of 0.5 mM ZnCl_2_ (+Zn). Blots were exposed to anti-OprD or anti-Hsp70 (loading control) antibody. OprD quantification was performed using ImageJ software. OprD band intensity was normalized to Hsp70 intensity and expressed as a percentage of the condition of the absence of Zn; (**C**) Amount of *oprD* mRNA analyzed by quantitative RT-PCR in the *ΔczcRS* and the *ΔczcRS;hfq^−^* mutants cultivated in the absence or presence of 0.5 mM ZnCl_2_ as indicated. The amount of mRNA is represented relative to the WT strain cultivated in the absence of metal. Statistics are indicated, using *p* values of <0.05. (*). Statistically, there is no difference between *ΔczcRS* (-Zn) and *ΔczcRS;hfq^−^* (-Zn) as well as between *ΔczcRS;hfq^−^* (+/-Zn). Experiments were performed in triplicate on three independent occasions. Error bars represent the standard deviations of three independent determinations.

**Figure A1:**
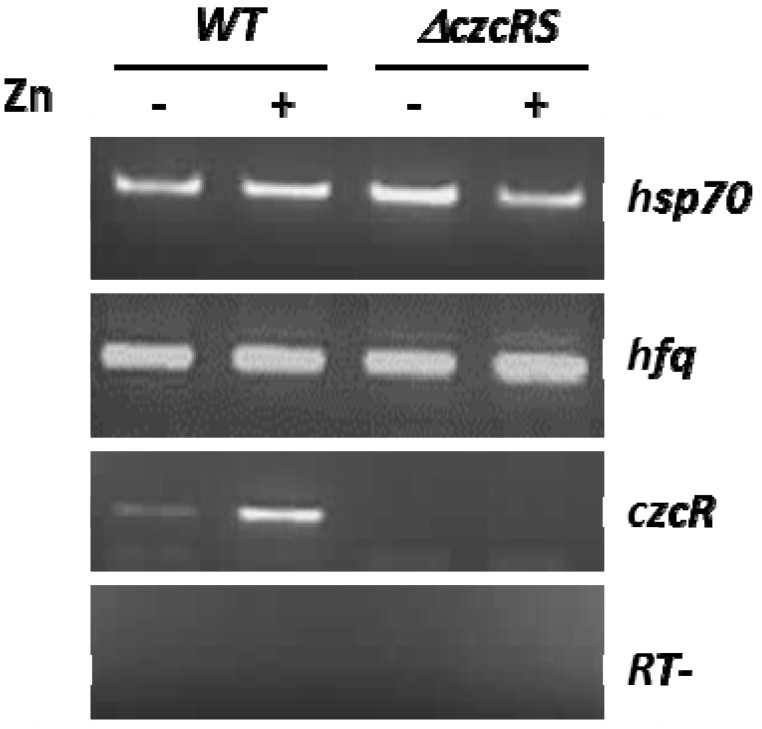
Hfq expression is not affected by the presence of Zn. Transcription of *hsp70*, *hfq* and *czcR* genes in the WT and the *ΔczcRS* mutants grown in LB medium in the absence or presence of 0.5 mM Zn as indicated, analyzed by semi-quantitative RT-PCR. Negative controls (*hsp70* gene) lacked reverse transcriptase (*RT-*).

The RNA chaperone Hfq is known to control the expression of several porins in Gram-negative bacteria, working mostly at the post-transcriptional level, on mRNA stability or translation [17,35]. We therefore decided to test the possible involvement of this protein in the repression of OprD upon Zn treatment. To this aim, an *hfq* mutant was created in the *ΔczcRS* genetic background to avoid the CzcR-mediated transcriptional repression. A slight growth delay was observed in the *hfq* mutants (*hfq^−^* and *ΔczcRS;hfq^−^*) in the presence of metal. This reflects the growth rate defect already detected in the *P. aeruginosa* *hfq* mutant [26]. The inoculum was therefore adapted (see experimental Section 2.3.) in order to reach the same optical density after 6h of growth (Figure 2A). As expected, the *ΔczcRS* mutant displayed a repression of OprD in the presence of 0.5 mM Zn, whereas the effect on OprD level was only very weak (5%) in the *ΔczcRS;hfq^−^* double mutant (Figure 2B). These observations suggested the possible involvement of the RNA chaperone Hfq in the control of OprD production under metal stress conditions. To investigate the contribution of Hfq in this process, we first determined the amount of *oprD* mRNA by quantitative RT-PCR. In the *ΔczcRS* mutant, addition of Zn induced a drop in *oprD* mRNA, while disruption of the *hfq* gene in this strain (*ΔczcRS;hfq^−^* mutant) totally abolished the decrease in the amount of *oprD* mRNA (Figure 2C). Zn treatment however did not affect the expression of *hfq*, as measured by semi-qRT-PCR (Appendix Figure A1). Altogether these results showed that, in addition to the transcriptional repression mediated by CzcR, Zn treatment induced an Hfq-dependent mechanism leading to *oprD* mRNA down-regulation.

### 3.3. Hfq is also Involved in the Negative Regulation of OprD Mediated by CzcR

**Figure 3:**
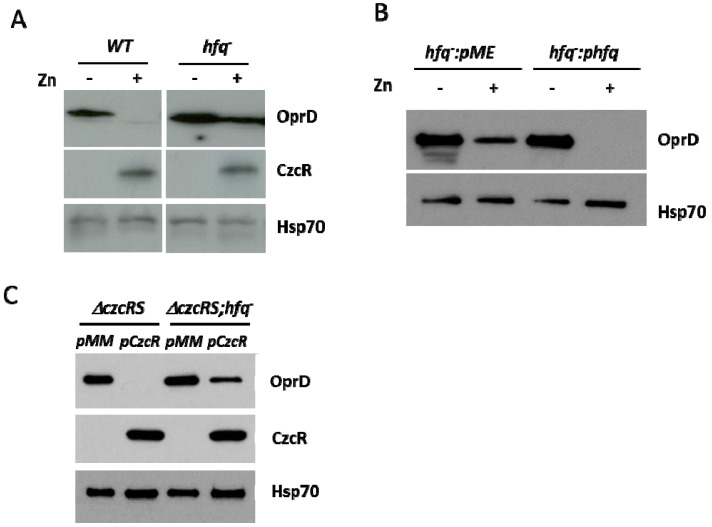
Repression of OprD requires Hfq. Immunoblot analysis on total protein extract of: (**A**) the WT strain and the *hfq^−^* mutant cultivated in the absence or presence of 0.5 mM ZnCl_2_ as indicated; (**B**) the *hfq^−^* mutant complemented with the empty plasmid (pME) or with the *hfq* gene (phfq) cultured in the absence or presence of 0.5 mM ZnCl_2_ as indicated; (**C**) the *ΔczcRS* and the *ΔczcRS;hfq^−^* mutants transformed with either an empty (pMMB66EH) plasmid (pMM) or a plasmid containing the *czcR* gene (pCzcR) under an IPTG inducible *taq* promoter. Cultures were grown for 6 hours in presence of 0.1 mM IPTG. Blots were exposed to anti-OprD, anti-CzcR or anti-Hsp70 for the loading control.

In the absence of CzcRS, we observed that Hfq might contribute to the negative regulation of OprD upon Zn challenge (Figure 2). To test whether Hfq contributes to CzcRS TCS function, we examined the amount of OprD porin in the WT strain and in the single *hfq* mutant. Surprisingly, while the CzcR protein was induced at the same level in both strains in presence of Zn, OprD repression is clearly impaired in the absence of Hfq protein (Figure 3A). This result suggested that either Hfq is the main OprD repressor in Zn conditions or CzcR-mediated regulation requires the RNA chaperone. Complementation of the *hfq^−^* strain with a plasmid containing a functional 6x His-tagged *hfq* gene led to a full restoration of OprD repression in the presence of Zn (Figure 3B), indicating that the observed result was not due to a polar effect caused by *hfq* deletion. The regulation of OprD in the control WT strain containing either the pME (empty plasmid) or the *hfq* gene (p*hfq*) was not affected (data not shown). Moreover, the fact that the CzcR protein is fully produced suggested that Zn recognition by the CzcS sensor protein and the phosphorelay leading to CzcR induction are not affected by the absence of Hfq.

We have previously noticed that artificial overexpression of the transcriptional regulator CzcR is able to repress *oprD*, even in the absence of the sensor protein CzcS and in the absence of Zn [7]. We wondered whether in this situation negative regulation also required the Hfq protein. To this aim, we used the pCzcR plasmid containing the *czcR* gene under the control of an IPTG-inducible promoter. In the absence of Zn overexpression of *czcR* yielded complete repression of OprD in a *ΔczcRS* background, while only a minor effect was visible in the absence of Hfq (*ΔczcRS;hfq^−^* background) (Figure 3C). This indicates that full repression, mediated directly by CzcR, is dependent upon the Hfq protein even in the absence of Zn challenge.

### 3.4. Hfq is Necessary for Localization of CzcR to the oprD Promoter

**Figure 4:**
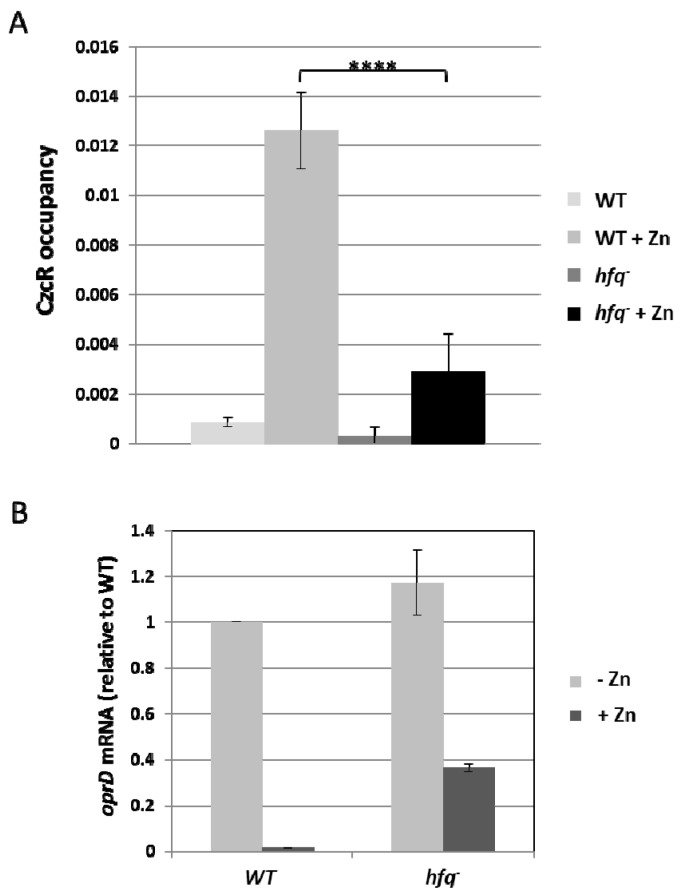
Hfq is necessary for the binding of CzcR to the *oprD* promoter. (**A**) Chromosome immunoprecipitation experiment performed on the WT strain and the *hfq* mutant using anti-CzcR antibody. The experiment was performed on cultures grown in the absence or presence of 0.5 mM ZnCl_2_, as indicated. Results represent the average of 3 independent experiments and standard deviations are indicated. Statistical analysis with the Student test gave a *p* value below 0.0001 (****); (**B**) Amount of *oprD* mRNA analyzed by quantitative RT-PCR in the WT and the *hfq^−^* mutants cultivated in the absence or presence of 0.5 mM ZnCl_2_ as indicated. The amount of mRNA is represented relative to the WT strain cultivated in the absence of metal. ChIP (chromosome immunoprecipitation) was performed in triplicate on three independent experiments. Error bars represent the standard deviations of three independent determinations.

In order to investigate the role of Hfq in CzcR-mediated transcriptional repression, we evaluated the DNA-binding efficiency of CzcR to the *oprD* promoter. To do so, we performed a ChIP (chromosome immunoprecipitation) experiment using anti-CzcR antibody. Interestingly, addition of Zn caused a clear localization of CzcR to the *oprD* promoter in the WT strain compared to the *hfq* mutant (Figure 4A). The weak occupancy of *oprD* promoter by CzcR observed in the *hfq^−^* strain is not due to a defect in *czcR* expression since the protein is produced to a similar level than in WT even in the absence of Hfq (Figure 3B). Furthermore, the fact that the level of transcriptional repression (Figure 4B) perfectly matches with the localization of CzcR on the *oprD* promoter (Figure 4A) suggests the requirement of RNA chaperone Hfq for the complete binding of CzcR to *oprD*, leading to transcriptional repression of the oprD porin.

### 3.5. Is Hfq also Involved in the Repression Mediated by other Transcriptional Regulators?

**Figure 5:**
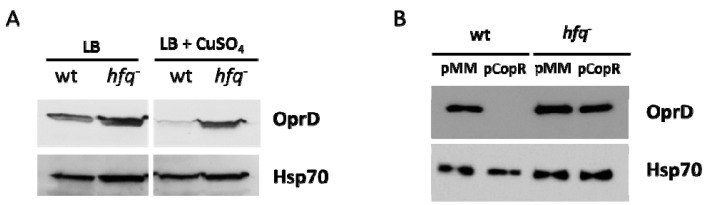
CopR requires Hfq for the repression of OprD in the presence of Cu. Immunoblot analysis of OprD porin on total protein extract of: (**A**) the WT and the *hfq^−^* mutants cultivated for 6 h in the absence or presence of 1 mM CuSO_4_; (**B**) the WT and the *hfq-* mutant complemented with either an empty plasmid (pMMB66EH) or a plasmid containing the *copR* gene under an IPTG inducible *taq* promoter. Cultures were grown for 6 h in presence of 0.1 mM IPTG. Blots were exposed to anti-OprD or anti-Hsp70 (loading control) antibody.

We have previously observed that CopR, the transcriptional regulator of the CopRS TCS involved in the copper response, also repressed the transcription of the *oprD* gene [5]. To determine whether this negative regulation mechanism also depends on the RNA chaperone Hfq, we first evaluated the effect of CopR induction after 6 h of growth in the presence of Cu. Western blot analysis showed no OprD repression in the absence of Hfq (Figure 5A). In addition, similarly to the overproduction of CzcR (Figure 3A), the overproduction of CopR, was unable to decrease OprD level in the absence of Hfq (Figure 5B). These results highlight the importance of Hfq for successful gene repression mediated by, at least, two TCS transcriptional regulators.

### 3.6. Involvement of the Hfq Protein in Carbapenem Resistance

According to these results, Hfq appears to be an important player in *P. aeruginosa* carbapenem resistance. In order to confirm this, we determined the minimum inhibitory concentration (MIC) for imipenem of various mutants (Table 2). Zn or Cu increased the MIC higher than the imipenem C value (8 µg·mL^−1^), conferring resistance to *P. aeruginosa* only in presence of Hfq protein. As previously observed [7], the CzcRS TCS is also essential for the imipenem resistance mechanism induced by Zn as the *ΔczcRS* mutant remained susceptible (Table 2). Interestingly, Hfq is also important for basal imipenem tolerance. In the *hfq^−^* or *ΔczcRS;hfq^−^* strains, the MIC dropped significantly even in absence of metals. Hfq is therefore a crucial player in OprD porin regulation, modulating imipenem resistance as a consequence of metal response.

## 4. Discussion

**Figure 6:**
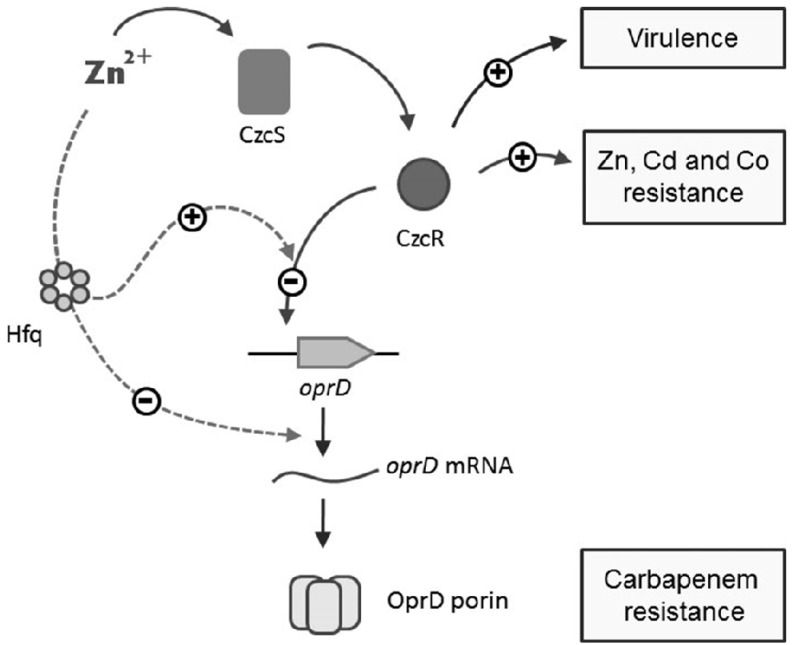
Co-regulation mechanism linking metal resistance, virulence and carbapenem resistance. Zn activates the CzcS sensor protein that will in turn activates the CzcR transcriptional regulator. CzcR induces the expression of CzcCBA efflux pump involved in Zn, Cd and Co resistance [7] and act positively on the virulence of *P. aeruginosa* [9]. CzcR is also implicated in the repression *oprD* transcription yielding to carbapenem resistance [7]. The RNA chaperone Hfq is necessary for the binding of CzcR to the *oprD* promoter leading to transcriptional repression. Additionally a Zn-inducible, Hfq-dependent mechanism decreases the amount of *oprD* mRNA yielding to an alternative pathway inducing carbapenem resistance in presence of Zn, and even in the absence of CzcR. Overexpression of the CopR protein, either by Cu or artificially, represses OprD only in the presence of the RNA chaperone Hfq. Additionally CopR is able to induce the expression of CzcR [5].

Carbapenems are an important class of antibiotics active against both Gram-negative and Gram-positive bacteria. They are often used as last resource for the treatment of *P. aeruginosa* infections. Carbapenem resistance in *P. aeruginosa*, however, is an important and worldwide problem with a resistance rate of about 20% in Europe [36]. These antibiotics penetrate the bacterium via the OprD porin, therefore the main and most frequent mechanism of resistance to this family of antibiotics is a decrease in the amount of OprD protein [6,37]. Several regulators and small molecules are known to modulate the expression of *oprD* (reviewed in [38]) and the post-transcriptional regulation of this porin has been speculated on for a long time [39], suggesting OprD as a highly regulated porin. In the present study we found that, in the presence of Zn or Cu, *oprD* is negatively regulated by two mechanisms dependent on the RNA chaperone Hfq (Figure 6). Hfq is a well-known RNA binding protein involved in post-transcriptional regulation via modulation of sRNA-mRNA interactions [17]. Since many sRNAs control gene expression in proteobacteria, mutations affecting the Hfq expression yield pleiotropic effects. In *P. aeruginosa*, mutations in Hfq strongly affect quorum sensing and virulence factors production [26].

**Figure A2:**
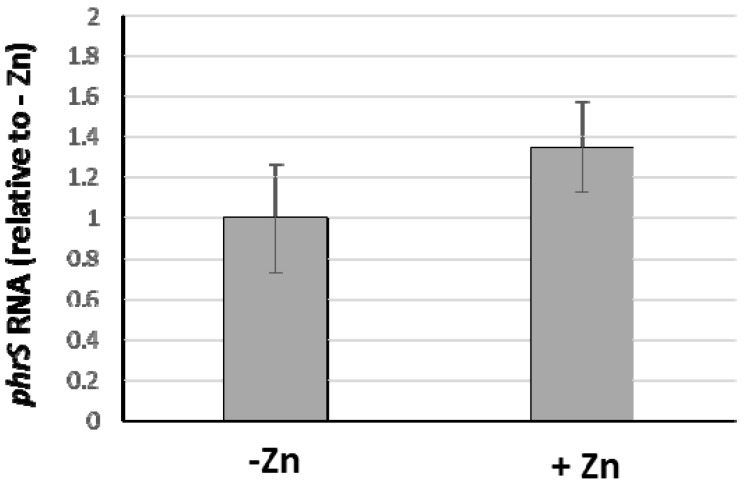
*phrS* is not modulated by Zn. Amount of *phrS* RNA analyzed by quantitative RT-PCR in the *ΔczcRS* mutant cultivated in the absence or presence of 0.5 mM ZnCl_2_ as indicated. The amount of RNA is represented relative to the strain cultivated in the absence of metal. Experiments were performed in triplicate on three independent occasions. Error bars represent the standard deviations of three independent determinations.

Since Zn treatment did not affect the expression of *hfq* (Appendix Figure A1), the Hfq-mediated regulation in the absence of CzcR might be due to a Zn-regulated sRNA. To date, the only known sRNA able to modulate *oprD* levels is *phrS* [40]. However, overexpression of this sRNA was shown to increase OprD protein levels [40] and Zn treatment failed to modulate *phrS* expression (Appendix Figure A2). To our knowledge, there are no sRNAs shown to be involved in *oprD* negative regulation. On the other hand, several studies have demonstrated that Hfq can bind directly to the target mRNA without the help of sRNA [41,42,43].

The transcriptional effect of Hfq on the regulation of *oprD* reveals an important function of this RNA chaperone targeting the functionality of two-component systems transcriptional regulators. In *P. aeruginosa*, some 130 TCS have been listed [11]; a huge number accounting for its great versatility and ability to sense and respond to environmental stimuli. These two-component systems usually consist of a sensor histidine kinase membrane-spanning protein involved in the detection of environmental stimuli. This sensor protein activates a response regulator by phosphorylation that, in turn, modulates gene expression by binding to the target promoters [44].

The data presented here demonstrate that Hfq is necessary for the binding activity of CzcR to the *oprD* promoter in the presence of Zn (Figure 4 and Figure 6), without affecting its expression or stability (Figure 3). The mechanism by which Hfq allows the binding of CzcR to the *oprD* promoter is not yet understood, but several possibilities could be investigated according to the known properties of this RNA chaperone [45]. Hfq is able to bind to DNA and organize the *E. coli* nucleoid [46], it could therefore be directly involved in the modeling of the *oprD* promoter, favoring CzcR binding. On the other hand, the possibility of an indirect effect on the regulation of *oprD* through the modulation of CzcR co-factor expression by a small RNA cannot be excluded.

We showed that Hfq is also necessary to the copper repression mediated by the CopRS TCS. In the presence of Cu, the CopR regulator represses *oprD* independently of CzcR [5]. We showed here that this activity is also Hfq-dependent (Figure 5). Interestingly in the absence of Hfq, the overexpression of CopR displayed no OprD repression (Figure 5B) while CzcR overexpression weakly decreased the amount of OprD protein (Figure 3C). This difference could be explained by the modest presence of CzcR on the *oprD* promoter even in the absence of Hfq (Figure 4A). This suggests that the Hfq requirement for repression mediated by TCS regulators might differ from one regulator to the next. The fact that Hfq is necessary for the repressor activity of two distinct TCSs suggests an important role of this RNA chaperone in the sensing and adaptation of *P. aeruginosa* to its environment. Consistently, this might explain the pleiotropic effects observed in the *hfq* mutant [25,26]. Taken together our data support the fact that the Hfq protein may represent a very interesting target for drug discovery [47]. Blocking Hfq functions will affect not only *P. aeruginosa* quorum sensing and virulence but also several TCSs involved in carbapenem resistance.

## 5. Conclusions

We previously observed that the environmental opportunistic pathogen *Pseudomonas aeruginosa* becomes more virulent and resistant to carbapenem antibiotic in the presence of metal pollutants such as zinc, or copper. The data presented here, bring new light on the mechanisms of metal inducible antibiotic resistance. We found that the transcriptional repression of OprD, involved in carbapenem resistance, requires the RNA chaperone Hfq. By revealing the underlying mechanism, we discovered that the metal specific transcriptional regulator CzcR requires Hfq to bind to the *oprD* promoter. Furthermore, we demonstrated that another transcriptional regulator from another two-component system also requires the Hfq protein to repress OprD in presence of metal. These results highlight how Hfq chaperone acts as an essential component of the environment-sensing in *P. aeruginosa,* liking metal detection to antibiotic resistance.

## Figures and Tables

**Table 1:** Strains and plasmids used in this study.

Strain or Plasmid	Relevant Characteristic(s)	Reference or Source
***Pseudomonas aeruginosa***		
Wild type (WT)	PAO1 wild type	WT, laboratory collection
Δ*czcRS*	PAO1 Δ*czcRS*	[5]
*hfq^−^*	PAO1 *hfq::aadA*, Sm^r^, Sp^r^	[26]
Δ*czcRS, hfq^−^*	PAO1 Δ*czcRS, hfq::aadA,* Sm^r^, Sp^r^	This study
***Escherichia coli***		
Top10	F-*mcrA* Δ(*mrr-hsd*RMS-*mcr*BC) Φ80*lac*ZΔM15 Δ *lac*X74 *rec*A1 *ara*D139 Δ(*araleu*)7697 *gal*U *gal*K *rps*L (Str^R^) *end*A1 *nup*G	Invitrogen
**Plasmids**		
pMMB66EH	Broad host range expressing vector, Cb^r^	[29]
pczcRWT pcopRWT	pMMB66EH derivative containing *czcR* gene, Cb^r^ pMMB66EH derivative containing *copR* gene, Cb^r^	[7] [5]
pME4510 pME-Hfq	Multicopy broad host range vector, Gm^r^ pME4510 derivative containing *hfq* gene under its own promoter, Gm^r^	[30] This study

Abbreviations of antibiotics. Sm: streptomycin; Sp: spectinomycin; Cb: carbenicillin; Gm: gentamycin.

**Table 2:** Minimum inhibitory concentrations for Imipenem (µg·mL^−1^) of the different mutants in the absence of metal or supplemented with 0.5 mM ZnCl_2_ or 2.5 mM CuSO_4_.

Strain	Medium Supplemented with:		
	No Metal	Zn	Cu
WT	1	16	>32
*ΔczcRS*	1	3	>32
*hfq^−^*	0.38	2	4
*ΔczcRS;hfq^−^*	0.25	0.38	2

## Appendix

**Table A1:** Primers used in this study.

Amplicon	Primer	Sequence (5′ to 3′)	Position	Length
***Coding region***				
*oprD*	oprD1	ATCTACCGCACAAACGATGAAGG	772	156
	oprD2	GCCGAAGCCGATATAATCAAACG	927	
*oprF*	oprF1	GGTTACTTCCTGACCGACGA	172	664
	oprF2	TCGGTGTTGATGTTGGTGAT	836	
*hfq*	*263*	GTCAAAAGGGCATTCGCTAC	3	176
	*331*	AGATCGCGTGCTTGTAAACC	178	
*hsp70*	406	CCACACCGTGATCGTCTATG	561	185
	407	CGCTTTCCTTCTTGAACTCG	745	
*czcR*	czcR1	GTCATCACCCGGACGCAGATCAT	502	153
	czcR2	GTAGCCGACGCCGCGAATGGTAT	654	
*phrS*	449	CAACTGGAGGCCATCAACAT	21	160
	450	CCTTGCGTGCTCTGTGTATC	180	
***Promoter***				
*poprD*	poprD R	CGCAGATGCGACATGCGTCA	−178	101
	poprD L	GGCGCTCCACTTCATCACTT	−77	
**Primers used for DNA cloning**				
*Hfq:6His*	609	GCCGAATTCGCGAAGCTGGAGCCATCTAC	−200	449
	667	GCCGGATCCTCAATGATGATGATGAT GATGAGCGTTGCCCGGCTCGG	249	
*Hfq insertion verification*	314	TGCCGTATACTGTCGCTCAG	PA4945	1390/3534 *
	315	ATTGAACAGGGTCGATTTGC	PA4944	

** Without and with aadA insertion cassette*.
